# A Review of Benign Hepatic Tumors and Their Imaging Characteristics

**DOI:** 10.7759/cureus.6813

**Published:** 2020-01-29

**Authors:** Stephen J Patacsil, Muhammad Noor, Alexander Leyva

**Affiliations:** 1 Radiology, AdventHealth Orlando, Orlando, USA

**Keywords:** hepatic hemangioma, focal nodular hyperplasia, hepatic tumor, hepatic adenoma

## Abstract

This paper concisely reviews the benign hepatic tumors most commonly encountered by clinicians. It includes the epidemiology, pathology, and imaging characteristics of hepatic hemangiomas, focal nodular hyperplasia (FNH), and hepatic adenomas (HAs).

## Introduction and background

Focal hepatic lesions are frequently encountered due to their wide variety of causes and incidental discovery on cross-sectional abdominal imaging. In this paper, we discuss the three most common benign hepatic tumors, namely hepatic hemangiomas, focal nodular hyperplasia (FNH), and hepatic adenomas (HAs). Along with a pertinent past medical history and physical examination, characteristic radiologic findings can often enable clinicians to noninvasively confirm these benign tumors and prevent unnecessary further workups.

## Review

Hepatic hemangioma

Hepatic hemangiomas are the most common benign tumors of the liver. In a large retrospective cross-sectional study consisting of 83,181 patients who had undergone abdominal CT and/or MRI scans, the prevalence of hepatic hemangiomas was found to be 2.5% [1]. Patients are usually middle-aged females, who are also more likely to present with symptomatic lesions.

Although often simply described as dilated vascular malformations, hepatic hemangiomas can demonstrate growth when exposed to increasing levels of estrogen and progesterone. There may be an association between the number of hepatic hemangiomas and lifelong exposure to estrogen [2]. They are typically solitary, range in size from several mm to more than 5 cm, and are found anywhere within the liver. Symptoms arise from distension of Glisson’s capsule or mass effect by lesions in the left hepatic lobe. Vascular spaces within the tumor may contain thrombi and subsequently develop calcifications.

Imaging Characteristics

**Figure 1:**
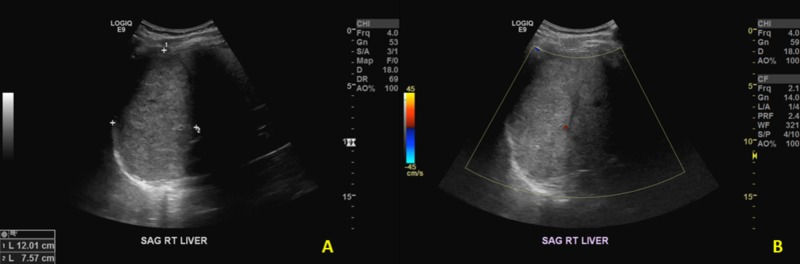
Liver ultrasound, sagittal view A. Sagittal ultrasound image of the liver demonstrating a hyperechoic mass within the liver with increased posterior through transmission. B. Doppler evaluation of this mass reveals no significant internal flow

**Figure 2:**
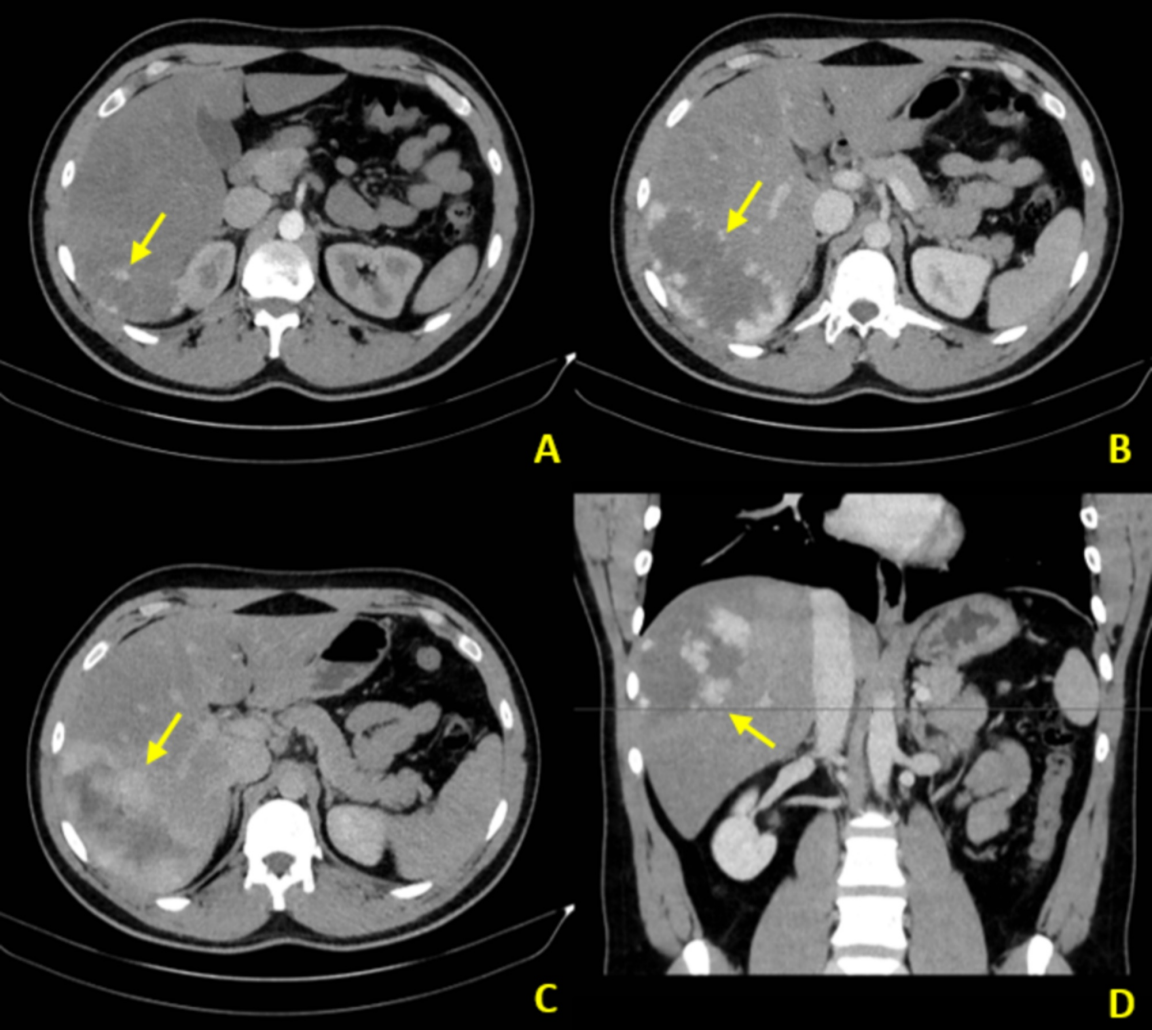
Triple-phase contrast-enhanced CT images CT: computed tomography A. Late arterial phase axial CT image in the same patient demonstrates discontinuous peripheral nodular enhancement (arrow) – classic for hepatic cavernous hemangiomas. B. Portal venous phase axial CT image at a more superior slice more clearly demonstrates the enhancement pattern (arrow). C. Venous phase axial CT image demonstrates slow centripetal filling (arrow). D. Portal venous phase coronal CT image provided to demonstrate location of hemangioma (arrow)

Although tumor calcifications can be demonstrated on plain abdominal radiographs, they are not specific for hepatic hemangiomas and therefore warrant additional imaging. Ultrasound may show a well-demarcated, homogeneous, and hyperechoic lesion with some variation (Figures 1A, 1B). Similarly, hepatic hemangiomas appear as well-demarcated, homogeneous masses on both non-contrast-enhanced CT and MRI scans. Contrasted studies depict discontinuous peripheral nodular enhancement and slow centripetal filling on delayed images (Figures 2A-2D) [3].

Focal nodular hyperplasia

FNH is the second most common benign tumor of the liver. The prevalence of FNH in the general population has been estimated to be approximately 3% in a necropsy study series [4]. Similar to hepatic hemangiomas, FNH has a tendency to occur in adult females, albeit asymptomatically.

Although its pathogenesis is not entirely clear, FNH is thought to develop following a hemodynamic disruption within the liver parenchyma. Our understanding of FNH as a hyperplastic adaptation is supported by an increased incidence in individuals with hepatic hemangiomas and extensive portal hypertension [5]. Large FNH may manifest with a centrally located scar on gross examination. Microscopically, FNH also undergoes a bile ductular reaction at the fibrous septa.

Imaging Characteristics

**Figure 3:**
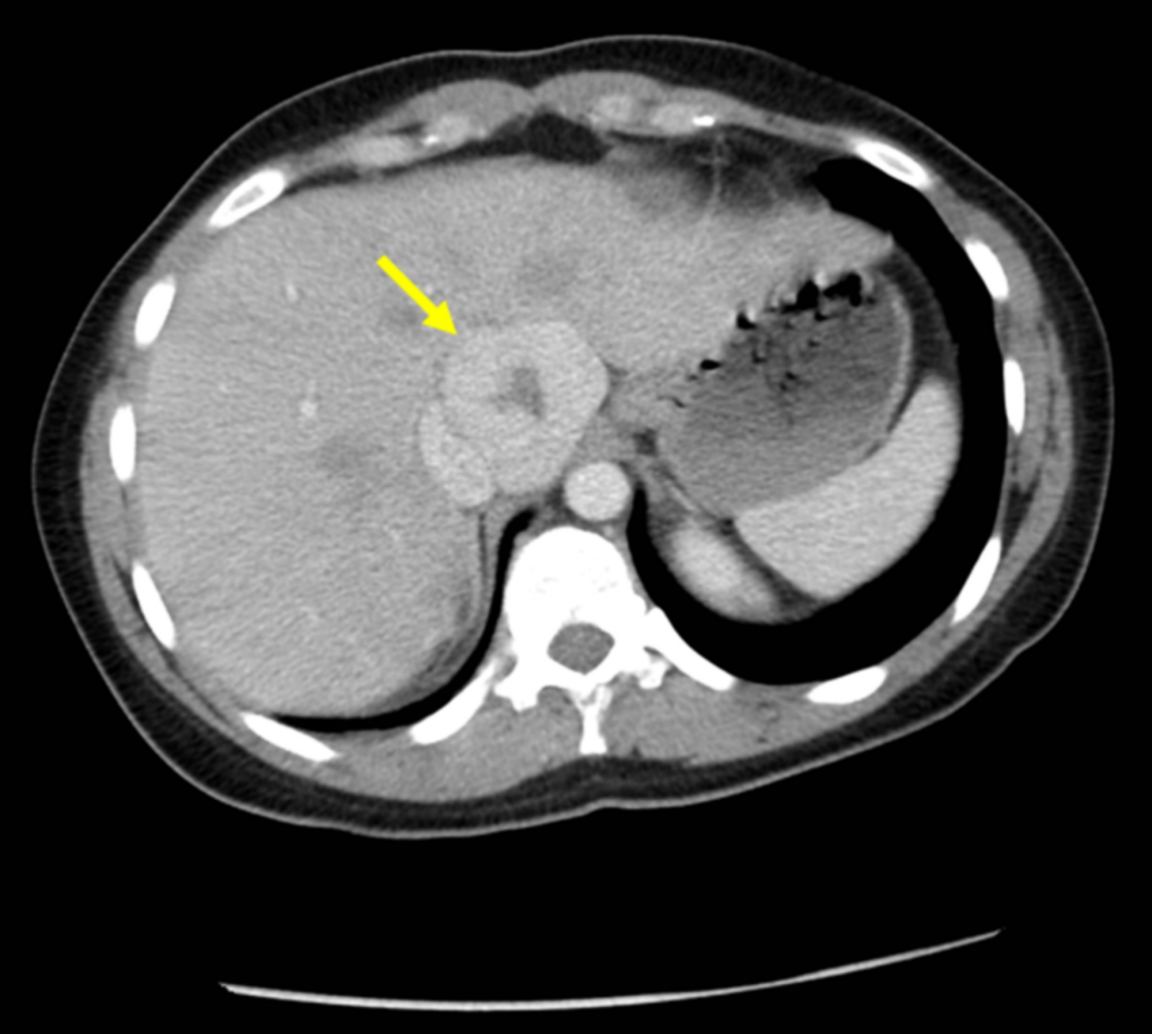
Contrast-enhanced abdominal CT CT: computed tomography Contrast-enhanced CT in portal venous phase demonstrates an enhancing mass with central non-enhancement. Imaging characteristics favor focal nodular hyperplasia

**Figure 4:**
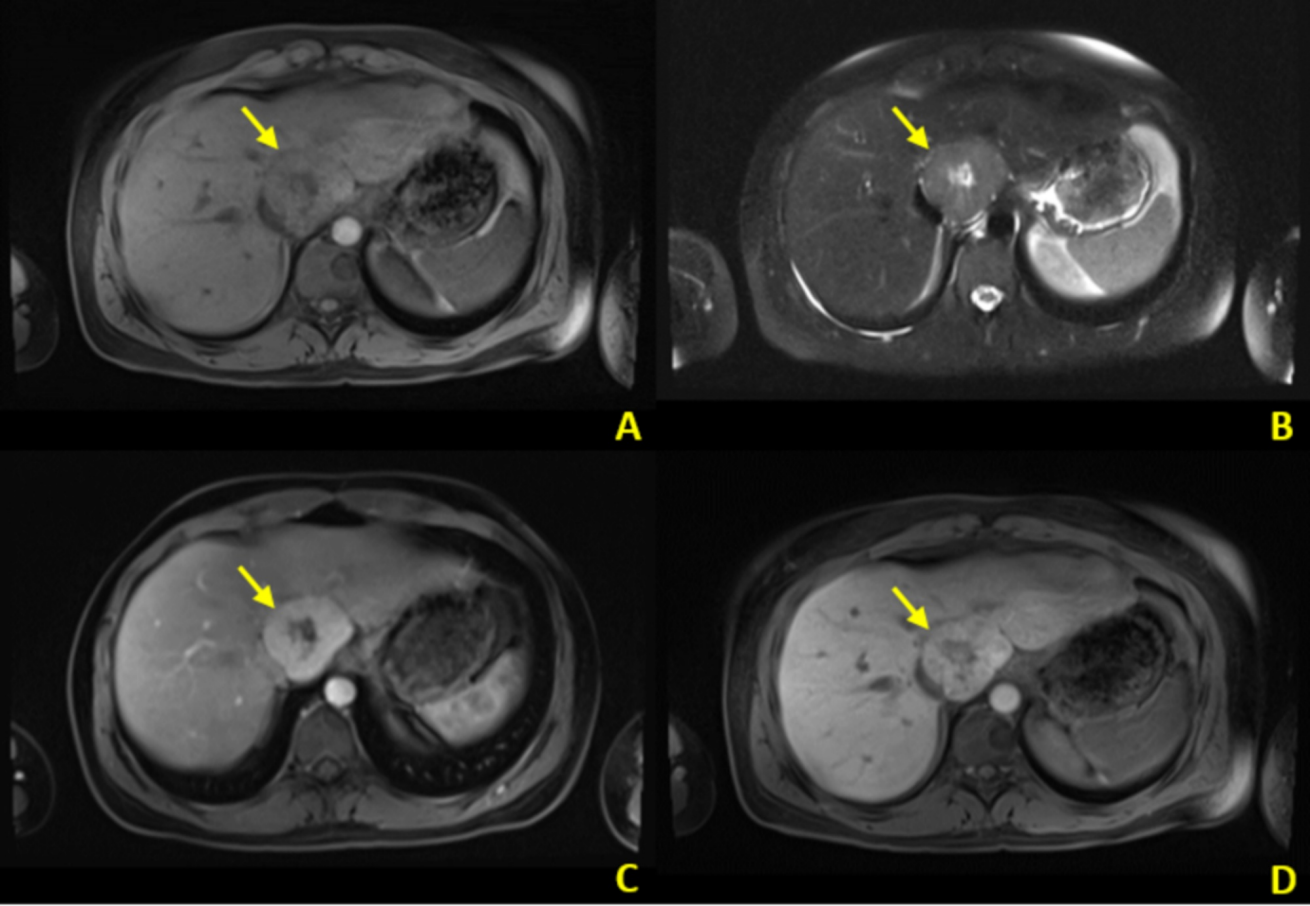
Contrast-enhanced abdominal MRI MRI: magnetic resonance imaging Contrast-enhanced (hepatobiliary agent) MRI of the same patient. A. T1 axial MR image demonstrates T1 isointense signal peripherally with a central focus of T1 hypointensity. B. T2 axial MRI demonstrates central T2 hyperintensity with relatively decreased signal in the periphery. C. Contrast-enhanced T1 MRI in bolus phase demonstrates peripheral enhancement of the mass. D. Contrast-enhanced T1 MR image in delayed/hepatobiliary phase demonstrates persistent contrast enhancement. Imaging characteristics favor FNH and confirm CT findings

FNH has variable echogenicity on ultrasound and infrequently demonstrates a stellate pattern in approximately 20% of patients [6]. CT scans of FNH feature hypodense lesions that display homogeneous arterial phase enhancement (Figure 3). Since many patients undergoing evaluation for FNH are reproductive-aged females, MRI is sometimes preferred over CT to avoid radiation exposure. Hepatobiliary MRI contrast agents, such as Eovist (Bayer AG, Leverkusen, Germany), reliably distinguish between FNH and other focal hepatic lesions since the former retain contrast in the hepatobiliary phase (Figures 4A-4D) [7].

Hepatic adenoma

HAs are the third most common benign tumors of the liver. Of the three benign hepatic tumors discussed in this paper, HAs have the poorest estimations of prevalence in the general population. The incidence of HAs in nonusers of oral contraceptives (OCs) is approximately one per million and increases nearly fourfold in female users of OCs [8]. The association of HAs with OCs is particularly strong in those who have been taking OCs for longer than 24 months. In addition to those taking long-term oral contraceptives, patients taking exogenous anabolic androgenic steroids or those with glycogen storage disease appear to have a relatively greater risk of developing HAs [9,10].

HAs histologically appeared as benign nodules of proliferating hepatocytes that may demonstrate steatosis and glycogen storage. HAs lack bile ducts and ductules, which helps to distinguish them from FNH. They ordinarily occur as solitary nodules with diameters ranging from less than 1 cm to greater than 15 cm. In a condition called hepatic adenomatosis, however, they are present in numbers greater than 10 [11].

Bleeding Risk and Malignant Transformation

Although the majority are asymptomatic, HAs do possess additional risks that need to be considered. HAs greater than 7 cm in diameter and those located directly underneath the hepatic capsule may spontaneously rupture and cause hemorrhage. Additionally, the development of hepatocellular carcinoma (HCC) in patients lacking a prior history of chronic liver disease has been documented [12].

Imaging Characteristics

**Figure 5:**
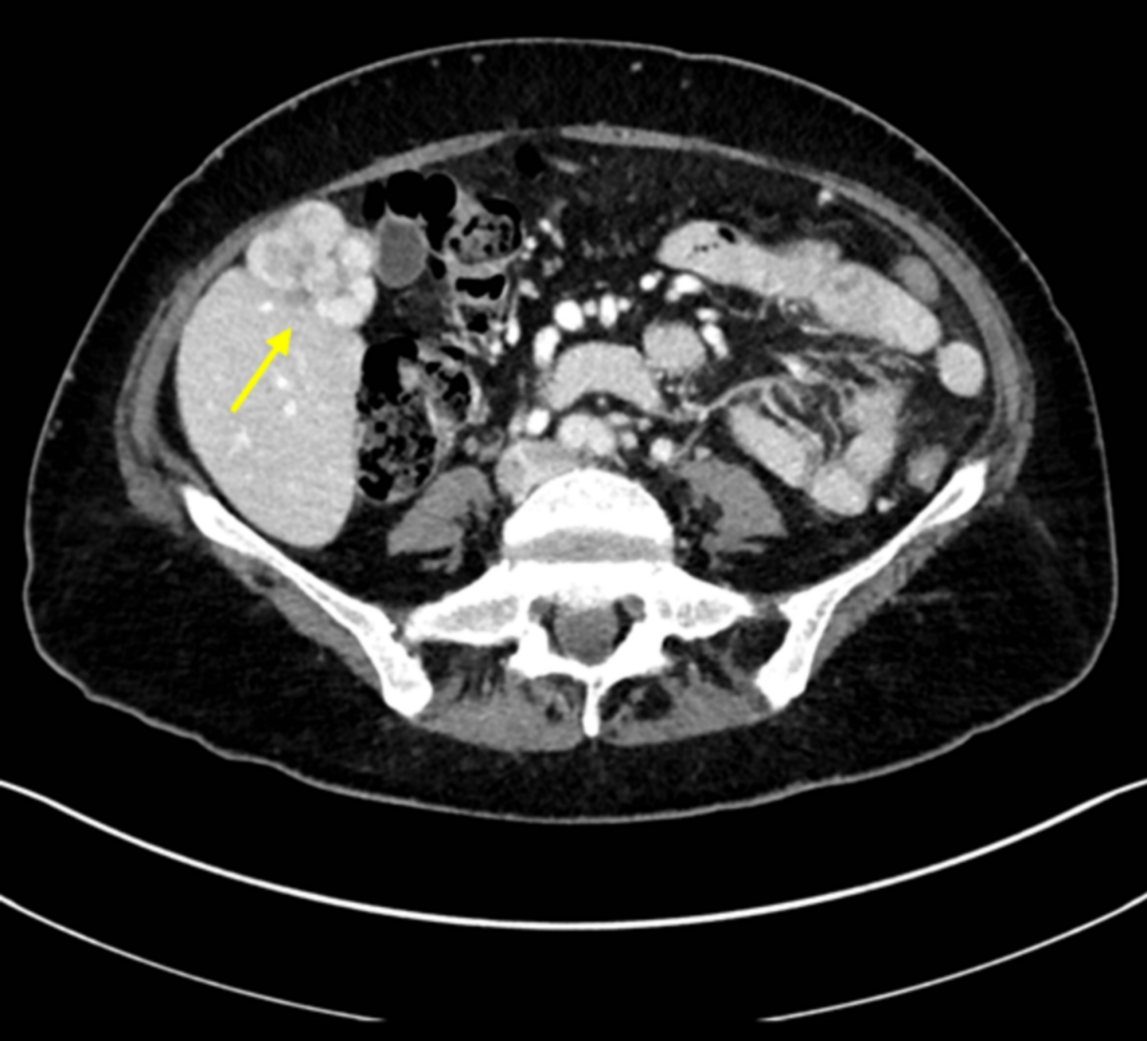
Contrast-enhanced abdominal CT CT: computed tomography CT image of a 38-year-old female on oral contraceptives. Portal venous phase contrast-enhanced axial CT image demonstrates a multilobulated mass with heterogeneous enhancement. This was a biopsy-proven adenoma

**Figure 6:**
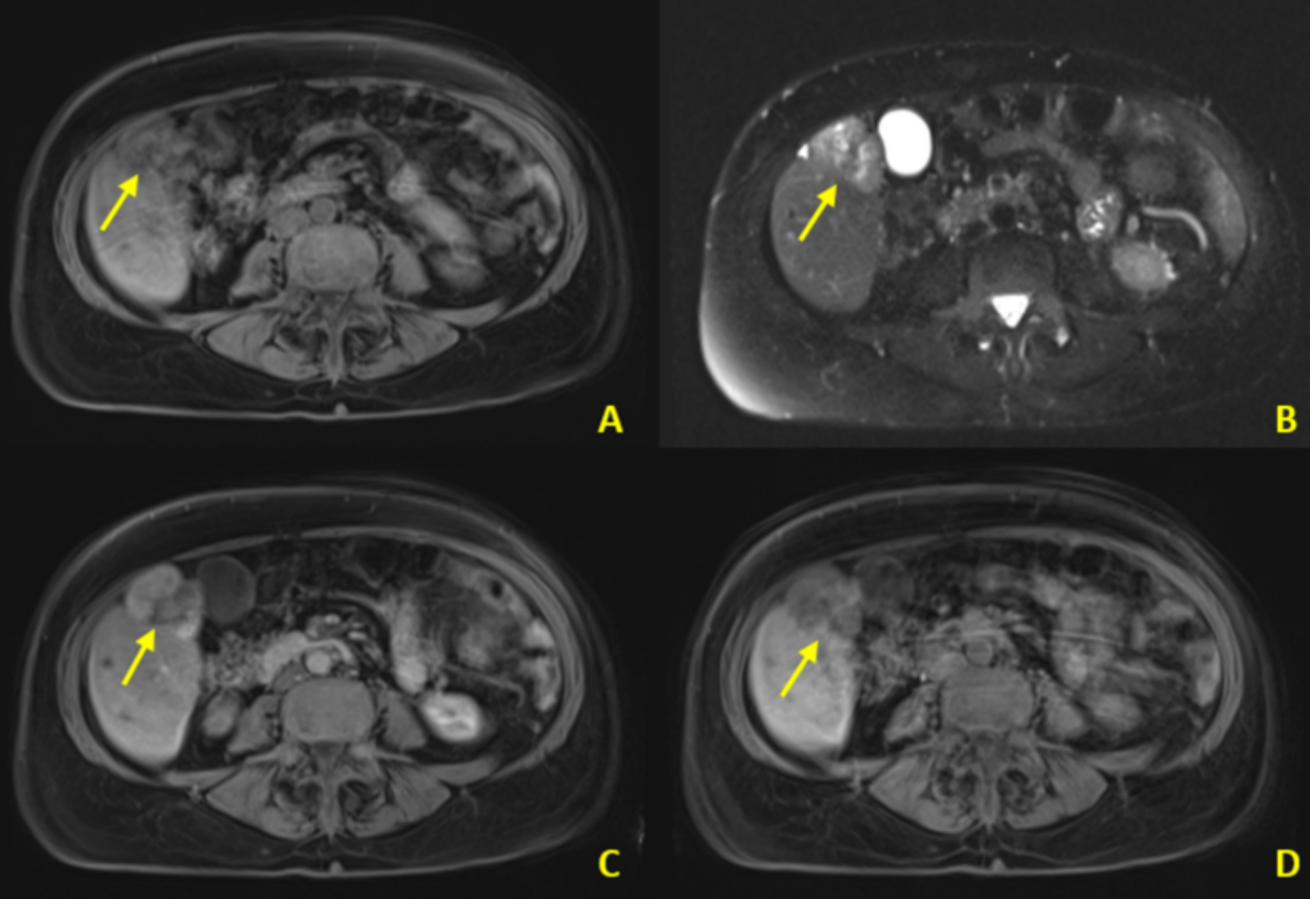
Contrast-enhanced abdominal MRI MRI: magnetic resonance imaging Contrast-enhanced (hepatobiliary agent) MRI from the same patient as in Figure 5. A. The mass demonstrates T1 hypointensity (arrow). B. There are scattered regions of T2 hyperintensity within the mass. C. Contrast-enhanced T1 bolus phase demonstrates early arterial enhancement. D. Contrast-enhanced T1 hepatobiliary phase image demonstrates relative wash-out of the mass compared to surrounding hepatic parenchyma. Enhancement pattern is consistent with a benign adenoma in this young female

On CT scan, HAs appear as well-demarcated, isointense lesions with peripheral enhancement (Figure 5). T1-weighted MRI shows hyperintense lesions due to hepatocellular steatosis and glycogen. HAs undergo early arterial phase enhancement and may take up Eovist during the portal venous phase, but they will not characteristically retain contrast during the hepatobiliary phase, which distinguishes HAs from FNH (Figures 6A-6D) [13].

Management

Individual malignancy risk will help to guide management for patients with any history of liver disease. The American Association for the Study of Liver Disease (AASLD) recommends surveillance ultrasound exams for patients with cirrhosis as they are at the greatest risk for developing HCC, which can deceptively demonstrate benign radiologic findings [14]. For these patients, subthreshold lesions of less than 10 mm in diameter require short interval follow-up to determine stability. Larger HCC lesions can often be noninvasively diagnosed because they are more likely to demonstrate abnormal enhancement patterns, extracapsular extension, and vascular invasion.

Hepatic hemangiomas less than 5 cm at initial presentation typically do not require follow-up imaging, as they often remain stable. Hepatic hemangiomas that demonstrate rapid growth may require arterial embolization or radiotherapy prior to resection [15]. FNH is similarly managed and resection is only necessary for symptomatic patients or if diagnostic imaging remains uncertain.

Since the growth of HAs is strongly correlated to the use of oral contraceptives and steroids, discontinuing these agents and follow-up imaging are appropriate for confirmed lesions of less than 5 cm. HAs greater than 7 cm are particularly prone to bleeding due to hypervascularization and fragile sinusoids and will require closer monitoring.

## Conclusions

Hepatic hemangiomas, FNH, and HAs are the three most common benign hepatic tumors. In practice, they are differentiated from each other based on the patient’s demographics, history of oral contraceptive or steroid use, and comorbidities such as glycogen storage disease and hepatic adenomatosis. Additionally, their characteristic radiologic findings allow clinicians to noninvasively diagnose and manage uncomplicated patients who possess no risk of malignancy. Patients with risk factors for HCC may require management as suggested in the referenced guidelines.
